# Experimental evolution suggests rapid assembly of the ‘selfing syndrome’ from standing variation in *Mimulus guttatus*

**DOI:** 10.3389/fpls.2024.1378568

**Published:** 2024-08-27

**Authors:** Sharifu K. Tusuubira, John K. Kelly

**Affiliations:** Department of Ecology and Evolutionary Biology, University of Kansas, Lawrence, KS, United States

**Keywords:** floral traits, mixed mating systems, *Mimulus guttatus*, selfing, outcrossing

## Abstract

Ecological and evolutionary changes are likely to occur rapidly when outcrossing populations experience pollinator loss. However, the number and identify of plant traits that will respond to this form of selection, as well as the overall predictability of evolutionary responses, remain unclear. We experimentally evolved 20 large replicate populations of *Mimulus guttatus* for 10 generations under three treatments: pure outcrossing, mixed mating (10% outcrossing) and pure selfing. These populations were founded from the same genetically diverse and outcrossing natural population. After 10 generations, all measured traits evolved with flower size, phenology, and reproductive traits diverging consistently among mating system treatments. Autogamy increased dramatically in the selfing treatment, but the magnitude of adaptation only becomes clear once inbreeding depression is factored out. Selfing treatment plants evolved reduced stigma-anther separation, and also exhibited declines in flower size and per-flower reproductive capacity. Flower size also declined in selfing populations but this was driven mainly by inbreeding depression and cannot be attributed to adaptation towards the selfing syndrome. Generally, the mixed mating populations evolved trait values intermediate to the fully selfing and outcrossing populations. Overall, our experimental treatments reiterated differences that have been documented in interspecific comparisons between selfing and outcrossing species pairs. Given that such contrasts involve species separated by thousands or even millions of generations, it is noteworthy that large evolutionary responses were obtained from genetic variation segregating within a single natural population.

## Introduction

Biologists have long been fascinated by plant mating systems and by the intricate relationships between mating strategies and the physical characteristics of plants (Barrett, 1998). Species with different pollinators exhibit predictable differences in floral traits such as red flowers for hummingbirds and white flowers for moths. These trait assemblies are often called “pollination syndromes” (Ornduff, 1969). Most Angiosperms are self-compatible in that individuals can reproduce by either self-pollinating ovules (selfing) or by accepting pollen from other individuals (outcrossing) (Richards, 1997) and the relative frequency of these outcomes is a critical aspect of the mating system. Species with a very low rate of outcrossing tend to have the selfing syndrome, a collection of traits such as highly reduced flowers that are adaptive with that mode of reproduction (Sicard and Lenhard, 2011). Selfing species have evolved from many distinct outcrossing lineages (Barrett et al., 1997, 2014; Jain, 1976; Stebbins, 2013; Zhong et al., 2019 *)*.

One of the great advantages of self-fertilization is that it provides reproductive assurance, allowing plants to produce seed when pollinators are unavailable (Baker, 1955; Lloyd, 1965; Busch and Delph, 2012). If an outcrossing but self-compatible population experiences a loss or substantial reduction in pollinator service, it should experience strong selection for more efficient selfing. Selfing syndrome traits are obvious candidates to evolve in response to this selection. These include flower size (Ruane et al., 2014), flower color (Sapir et al., 2021), floral scents (Adhikari et al., 2021), nectar volume (Wessinger et al., 2014), nectar sugar concentration (Perret et al., 2001), and herkogamy (the distance between anthers and stigmas, Herlihy and Eckert, 2007). These traits usually vary within populations and this variation typically has a genetic component (Charlesworth and Charlesworth, 1995). The existence of genetic variation indicates that a response to selection is possible, but it remains unclear how rapidly traits will change following pollinator loss/decline. It may be that only a subset of key traits respond in the short term followed by much more gradual evolution of other syndrome characteristics. Alternatively, strong selection might yield a highly multivariate short-term response strongly influenced by the genetic correlations among traits (Ivey and Carr, 2012), correlations that can change rapidly as the mating system changes (Kelly, 1999b).

In this paper, we use experimental evolution to test how a predominantly outcrossing population will evolve when confronted with an environmental change that necessitates high or complete selfing. The ancestral population for this experiment is a single genetically diverse sample of *M. guttatus* (yellow monkeyflower). Mating system traits exhibit high heritable variation in *M. guttatus* (Carr and Fenster, 1994; Robertson et al., 1994; Kelly and Arathi, 2003; van Kleunen and Ritland, 2004; Scoville et al., 2009). We monitor evolution in the rate of development, measures of flower size, stigma-anther separation, and the capacity for plants to reproduce by both outcrossing and selfing (autogamy). These traits exhibit pronounced and generally consistent differences between outcrossing and selfing species in the genus (Ritland and Ritland, 1989; Fishman et al., 2002) and also correlate with mating outcomes under field conditions (Hall and Willis, 2006; van Kleunen, 2007; Jordan et al., 2015; Shelton et al., 2024). Stigma-anther separation has been linked to outcrossing rate in numerous Mimulus species (Fetscher et al., 2002; Richardson, 2004; Carvallo and Medel, 2010).

The immediate evolutionary response to altered pollinator availability has been investigated in both experimental (Kofler et al., 2024) and natural populations (Acoca-Pidolle et al., 2023; Bishop et al., 2023). The present experiment was designed based on results from the previous study of mating system evolution by Bodbyl Roels and Kelly (2011). Those authors allowed replicate populations of *M. guttatus* to evolve with or without bumblebees and documented an immediate response: Populations denied bees evolved an increased capacity to set seed autogamously (within flower selfing). However, evolutionary changes in morphological features such as flower size were not clearly predicted by mating system treatment. The present experiment differs in both design and scale reflecting a number of “lessons learned” from the previous study.

**Figure 1:**
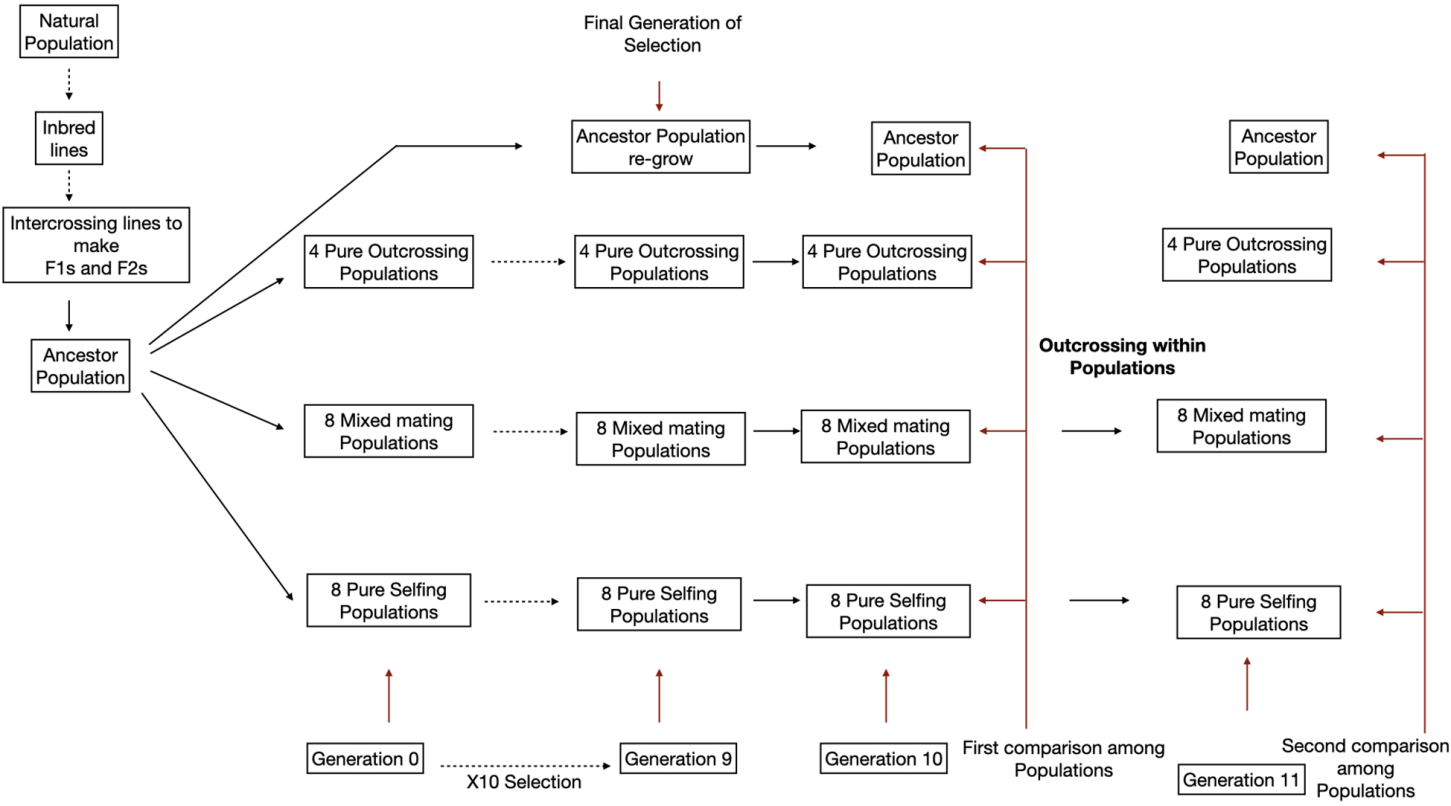
A schematic representation of the experimental setup. Evolutionary change was measured within four replicates for pure outcrossing, eight replicates with mixed mating (10% outcross and 90% selfing) and pure selfing. Black dotted lines indicate the passage of multiple generations while solid black lines indicate one generation. Solid brown lines identify comparisons.

Regarding design (**Figure 1**), we here include three treatments: outcrossing, selfing and mixed mating. The mixed mating category was included because pollinator declines may often be incomplete, allowing outcrossing but at a much-reduced rate. Second, we here replace the presence/absence of bees with direct control of the outcrossing rate. This change allows us to isolate the effect of mating system change from the multiple selective effects imposed by bees. Bumblebees carry pollen between plants (outcrossing) but can also cause a plant to self-fertilize by physically manipulating flower parts. Third, we raised the number of experimental populations from 4 to 20 which greatly increases our power to detect changes that occur in a consistent direction within mating system treatments (**Figure 1**). Bodbyl Roels and Kelly (2011) found that selfing greatly increases the variability among replicate populations (see also Busch et al., 2022), which was expected given that selfing amplifies stochastic changes in allele frequency (Caballero and Hill, 1992; Hartfield et al., 2017). Increased variability among replicates makes it more difficult to detect treatment effects (which here are the average across replicate populations within mating system treatments). Higher population replication within our mixed and selfing treatments anticipates the stochastic effect of inbreeding.

The inclusion of experimental crosses post-selection (**Figure 1**) is an essential design feature because a directional difference among treatments – say that mean flower size declines in the selfing but not outcrossing populations – is not compelling evidence of adaptation. When populations experience a change in mating system, trait means will change for two reasons. The most immediate effect is that inbreeding increases the average homozygosity within the population and trait means change due to inbreeding depression. Inbreeding depression is usually considered in relation to fitness (Charlesworth and Charlesworth, 1987), but experimental studies in many species have shown that it can influence many aspects of the phenotype. Relevant to mating system evolution, inbreeding has been shown to change traits like flower size and days to flower (without any selection) in many plants (Andersson, 1996, 2012; Rathcke and Real, 1993; Willis, 1996; Shaw et al., 1998; Kelly and Arathi, 2003; Kariyat et al., 2021; Qu et al., 2023). To identify the second (and perhaps more interesting) cause of changes in traits – change in allele frequencies driven by natural selection – we need to factor out inbreeding depression effects. As in Bodbyl Roels and Kelly (2011), we apply controlled crosses within evolved populations at the end of the experiment. This enables comparison among plants where difference can be attributed adaptation, that alleles favorable under the new mating system have increased in frequency.

## Materials and methods

### The plants

*Mimulus guttatus* (syn *Erythranthe guttata*; 2n = 28; Phrymaceae) is a model for plant genetics and mating systems evolution (Wu et al., 2008). We founded experimental populations by randomly pairing 124 highly inbred lines (Troth et al., 2018). The inbred lines were made by single seed descent (6-12 generations) from a large, random selection of field plants from one natural population of *M. guttatus* on Iron Mountain in Oregon (44°24′03″N, 122°08′57″W (Willis, 1993; Arathi and Kelly, 2004)). Reciprocal crosses between paired plants produced 62 F_1_ families. The F_1_ plants were randomly paired and crossed producing F_2_ seeds. F_2_ seed from all families was combined into a common pool and this pool (our “Ancestral population”) was used to establish 20 experimental populations. We created replicate populations within three treatments: four populations for pure outcrossing, eight populations for mixed mating (10% outcross and 90% selfing) and eight populations for pure selfing.

### The treatments

In each generation, for a total of ten generations (generations 0 through 9 in **Figure 1**), plants were grown following the same protocol. Each of the 20 populations was founded by 30 mg of seed distributed uniformly over the soil surface of a tub. The tubs were contiguous growth arenas (30.5 x 38.1 cm in area) with soil depth of 5 cm. Each tub could draw water through two holes in the bottom. The soil was kept fully hydrated for the first 11 days with a combination of bottom water and top misting. Tubs were rotated every other day, and all populations were treated equivalently in terms of growth conditions throughout the generation. On day 14, fertilizer (250 ml of Jack’s blossom booster (10-30-20) in 25 gallons of water) was applied by bottom watering. There was no further watering until day 21, when all tubs received bottom water for one hour. Flowering typically began around days 21-23. A progressive drought regime was initiated after day 21. Each tub received 1000 ml of water on days 28 and 32, then 500 ml on days 36 and 39, and finally 250 ml on day 42. This progressive drying mimics field conditions where nearly all plants eventually die of desiccation.

In the outcrossing treatment, we enforced outcrossing using hand pollination. All plants flowering on days 24-25 within the outcrossing treatment populations were hand pollinated, randomly choosing plants as pollen donors or recipients. Approximately 60% of the plants had at least one flower by days 24-25 and only one flower was used per plant (an average of about 280 flowers per population). Any incidental self-pollination in outcrossing treatment populations should be quantitatively insignificant because *M. guttatus* plants from Iron Mountain produce very few seeds from autogamy in the first 24 hours of the floral lifespan even when there is no competing cross-pollen (see **Figure 2** in Arathi et al., 2002). In the outcrossing treatment, receiving flowers had opened less than 8 hours before delivery of a saturating dose of cross-pollen via forceps (anthesis occurs between 4am and 8am and hand pollinations were conducted between 8am and noon on the day of anthesis). At the final harvest on days 45-47, only the hand pollinated fruits were collected and all seed was subsequently combined within each population.

**Figure 2:**
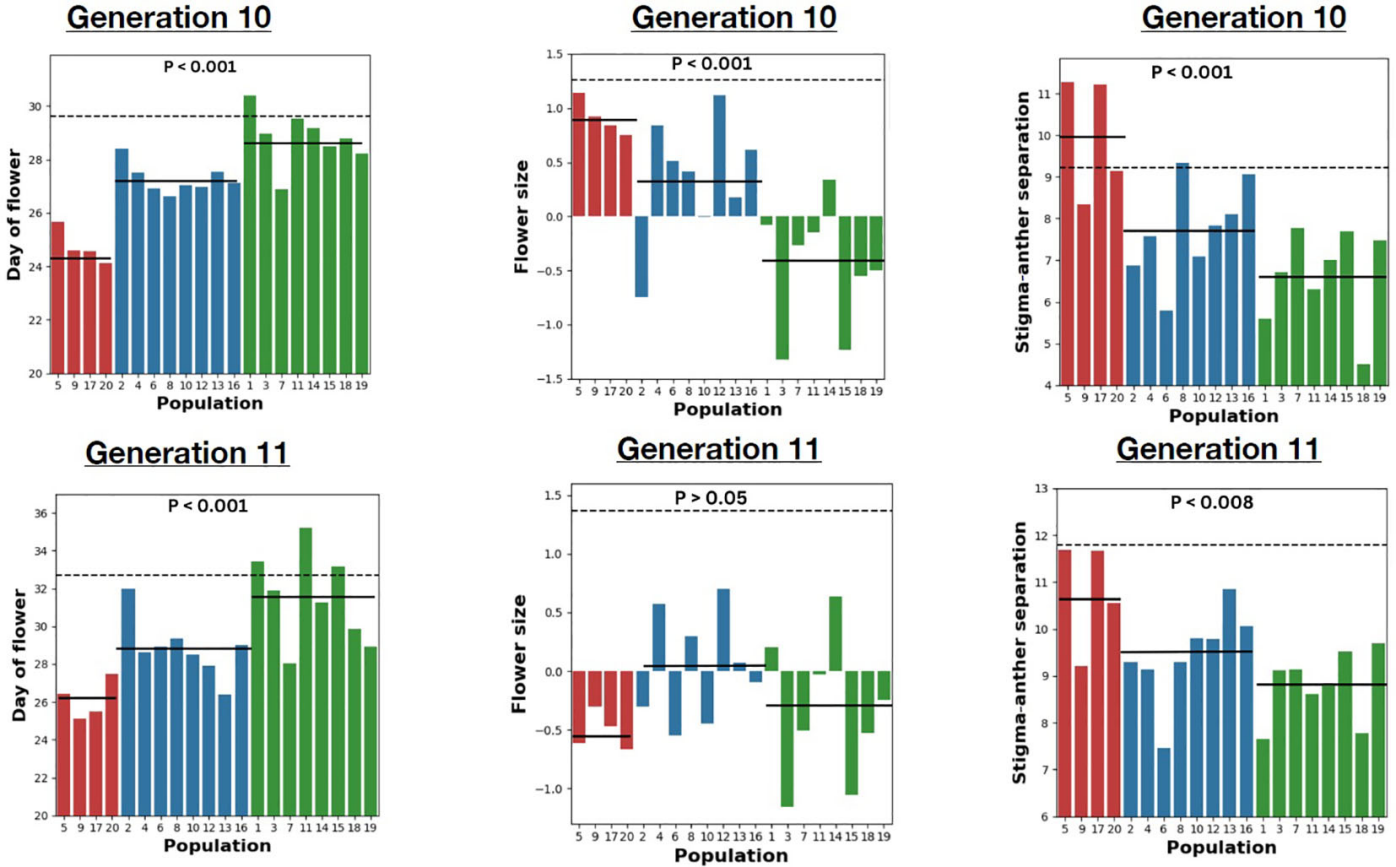
The mean for three traits in generations 10 and 11, respectively. Left: Day of flower (in days). Middle: Flower size measured as PC1. Right: Stigma-anther separation (hundredths of an inch). In all panels, red is outcrossing, blue for mixed mating, and green for selfing populations. The horizontal line dashed line indicates ancestral mean while solid line indicates the mean for the treatment. The p-values are from the Mating system treatment test of the nested ANOVA applied to each measurement (**Supplementary Table 4**).

In the mixed mating populations, 40 plants were randomly selected to receive hand pollination from another flowering plant on day 26-27. One flower was selected as either a pollen donor or recipient per plant. As described in the outcrossing treatment, the pollination was done in the morning hours between 8am and 12 pm on the select days. The hand pollinated fruits were marked to distinguish them from selfed fruits which were continuously produced until the end of the generation (routinely greater than 5 per plant). At the final harvest (days 45-47), we collected the hand pollinated fruits into one envelope and all others (those produced by selfing) into another. To make the founding seeds for each mixed mating population, we sampled 3mg from the hand-pollination collection and 27mg from the selfed collection (which enforces the 90% selfing condition for this treatment).

In the pure selfing populations, the plants were left undisturbed until the end of the generation. There was no opportunity for outcrossing in the pollinator free environment because the pollen is too large for air transport (and there is no wind anyway). All seeds were collected from the pure selfing populations (one pool per population). As described in the mixed mating populations, the number of selfed fruits per plant varied although it was routinely greater than 5 per plant across populations. At the final harvest (days 45-47) in each generation, all fruits were collected and the seeds combined.

In generation 9, we regrew 60 plants from ancestral seed pool and randomly paired and intercrossed these individuals to make 100 families. As described below, seeds from these families were grown alongside the descendant populations (Generations 10 and 11 in **Figure 1**). This “refresher generation” for the ancestral population eliminates any effects of seed age on phenotype (Franks et al., 2007).

### Phenotypic measurements on inbred and outbred plants

The progeny from the last generation of selection (Generation 10) were grown simultaneously with the ancestral population to maturity without selection. We obtained phenotypic measurements from 1409 plants (217 from the ancestral population and about 60 from each experimental population). On the day that each plant opened its first flower, we recorded the day of flowering and the length of the widest leaf. On the first two flowers produced by each plant, we recorded corolla width and length, pistil length and anther length (Fishman et al., 2002). Stigma-anther separation was the difference between pistil and anther lengths. For analysis, we averaged measurements across the two flowers per plant. The 3^rd^ flower on each plant was hand pollinated using a saturating amount of pollen from IM767. IM767 is an inbred line derived from the Iron Mountain population with high pollen viability. We grew hundreds of IM767 individuals simultaneously with Generations 10 and 11 to provide a source of genetically identical pollen. Finally, we marked the 4^th^ flower produced by each plant and allowed it to set seed via autogamy. We eventually collected all seeds from these 3^rd^ and 4^th^ flowers for counting and/or weighing.

Variation in seed produced through autogamy involves two components. Many plants fail to produce any seed. Those that set seed exhibit approximately log-normal variation in counts. Considering fruits where we both counted and weighed seed, we find a strong allometric relationship between mass and count (**Supplementary Figure 1**): *Log_10_(mass) = -0.992 + 0.775 Log_10_(count*). The slope (0.775) is positive but substantially lower than 1, indicating that individual seed mass declines as seed number per flower increases. Given these results, we decided to analyze autogamy as two components. The first is binary (0 or 1) based on whether a plant sets any seed at all (referred to as Prob of self-seed). Second, for plants that set self-seed, we log-transformed the count and denote this value as Log10(self-seed>0). With hand pollination, nearly all plants produced seed, again with approximately log-normal variation in both mass and counts. We calculated Log(mass+1) as reproductive capacity.

Alongside the plants used to take measurements in Generation 10, we grew 64 additional plants per population. These were used as pollen recipients (dams) for crossing from a randomly paired plant from the measured set within each population. The purpose of these crosses was to create outbred seed within each population for growth and measurement in Generation 11 (Kelly, 2005). The Generation 10 plants in the ancestral and outcrossing populations were already fully outbred, but plants within mixed mating and selfing populations were either partially or highly inbred. In Generation 11 (as shown in **Figure 1**), we grew plants from the ancestral population along with individuals from each descendant population (same genotypes grown in Generation 10). These descendant populations were used to generate outbred seed from each population. In addition, we obtained measurements on the same traits as in Generation 10 on a total of 1260 plants.

**Figure 3:**
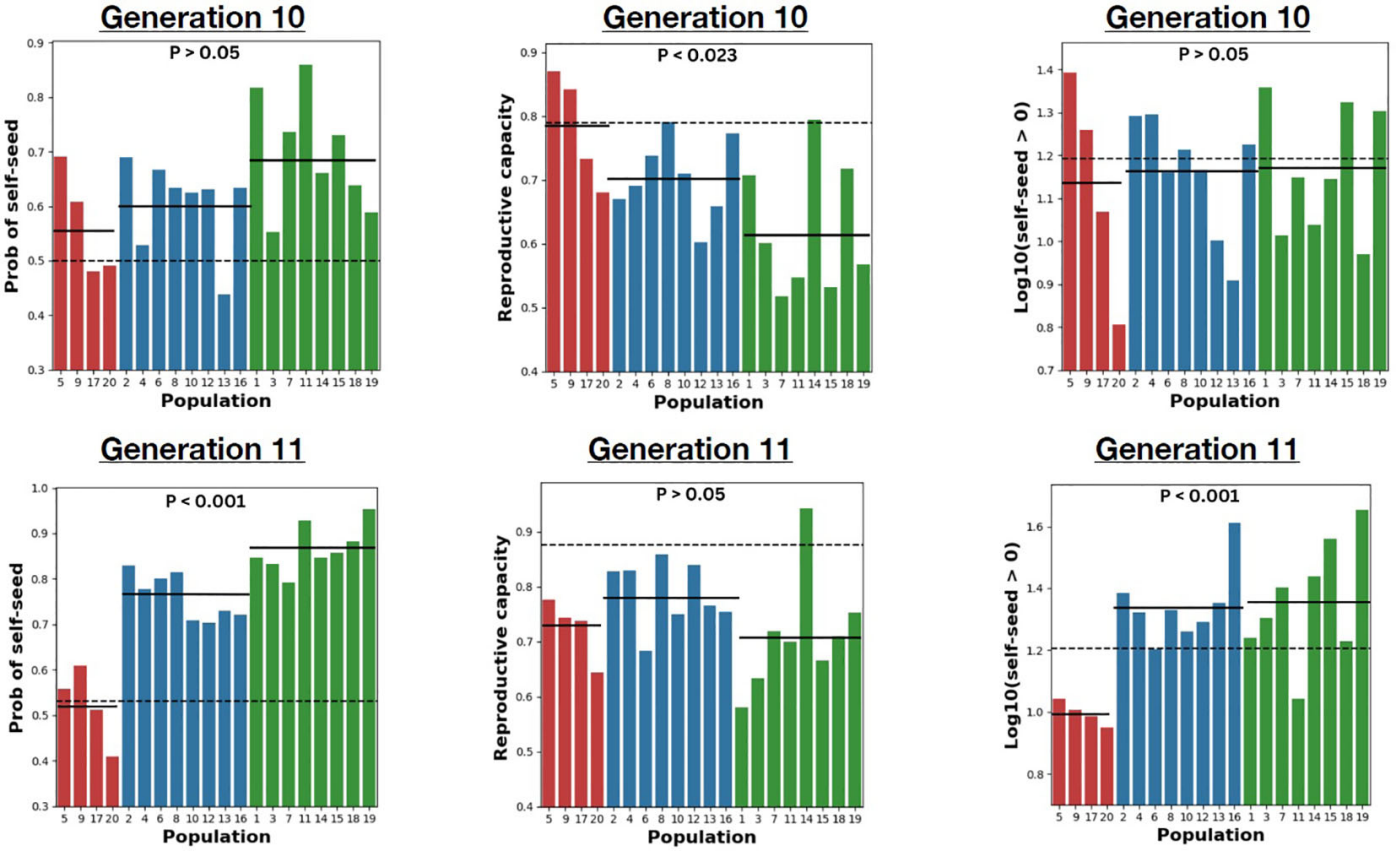
The mean for three fitness components in generations 10 and 11, respectively. Left: Probability of setting seed by selfing. Middle: Reproductive capacity (in Log(mg)). Right: Log10 (the number of seed set by selfing). In all panels, red is outcrossing, blue for mixed mating, and green for selfing populations. The horizontal line dashed line indicates ancestral mean while solid line indicates the mean for the treatment. The p-values are from the Mating system treatment test of the nested ANOVA applied to each measurement (**Supplementary Table 4**).

**Figure 4:**
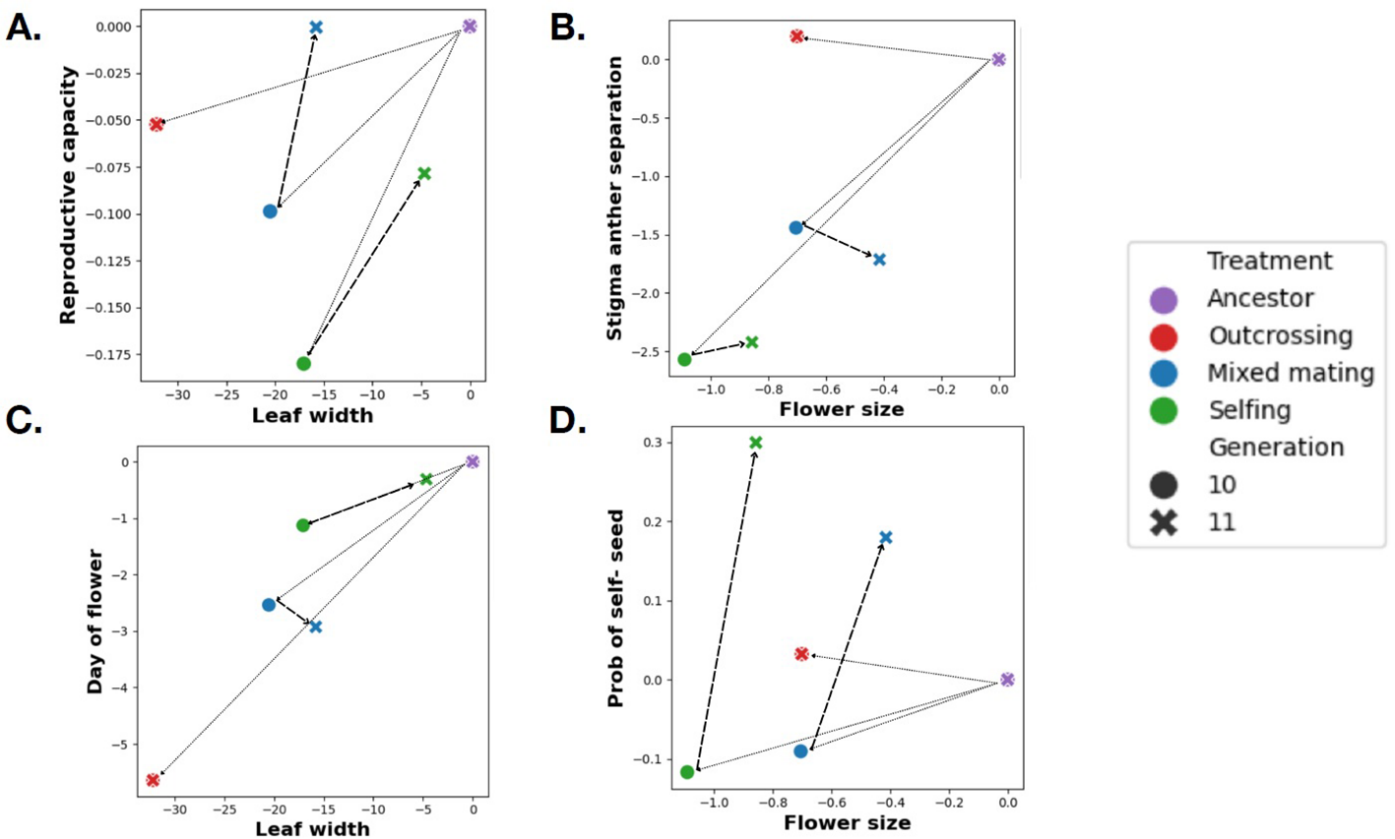
Bivariate plots showing changes in trait means from the Ancestor to Generation 10 (fine lines) and subsequently to Generation 11 (dashed lines with terminal arrow). The change in trait means indicates a shift from the effect of inbreeding depression to the effect of allele frequency changes. **(A)** Relationship of reproductive capacity and leaf width. **(B)** Relationship of stigma-anther separation and flower size **(C)** Relationship of day of flower and leaf width **(D)** Probability of setting seed by selfing and flower size.

Generation 10 was initiated on April 4, 2023, generation 11 on August 15, 2023. Each generation was grown as two distinct “cohorts” with seeds distributed to soil 14 days apart (dates above refer to start of first cohort). Within generations, populations were balanced across cohorts. We also grew a collection of Generation 10 seeds from each population simultaneously with the outbred plants of Generation 11 in cohorts 3-4. The balance across cohorts within generations is important to statistically factor out any effect of cohort on trait means. Cohort is included as a causal factor in the ANOVA models (described below) because there are seasonal effects on plants grown in the greenhouse. The within generation analyses (e.g. **Figures 2**, **3** below) use only the plants within that generation, while the integrated analysis (**Figure 4** below) considers all plants grown across all four cohorts.

### Statistical analyses

First, to test whether experimental populations diverged from the ancestral population, we applied one-way ANOVAs with each trait as the response and population as the factor. We used Tukey *post hoc* tests to identify which population means differed significantly from each other. Next, we excluded the ancestors to test for differences among descendant populations. To test whether experimental populations diverged from each other, and whether divergences were predicted by treatment, we applied a nested ANOVA (population within treatment) to each trait measured in Generation 10. Cohort was included as a random factor. We fit a total of 9 Nested ANOVAs to consider all the traits, with population nested in treatment. The same analysis was done on Generation 11 plants. Finally, we analyzed the data from Generations 10 and 11 simultaneously. To distinguish changes due to allele frequency from the direct effects of inbreeding, we used regression with inbred/outbred status of each population included as a covariate. This model also included growth cohort as a categorical predictor. The dependent variable was each trait, independent variables included inbred/outbred status as a covariate. After inspecting the floral trait data, we applied principal component analysis (PCA) to corolla width, anther length, corolla length and pistil length using the correlation matrix. One of our response variables, whether plants produced any seed by selfing, is binary and not continuous. This may cause our ANOVA to be underpowered. For this variable, we also performed Generalized linear model fits (logistic regression) with a binary response and with mating system and cohort included as categorical predictors. All statistical analysis was done using Minitab version 20 (Alin, 2010).

## Results

### Patterns of variation in traits and fitness components

The number of plants that progressed to flower varied among populations, and within population through time, but the average was appoximately 400. Total seed production was always much greater than the 30mg required, usually by more than tenfold (>300mg seed in total). For the plants grown and measured in generations 10 and 11, the means and standard deviations (SD) of each trait in each population are reported in **Supplementary Table 1**. As expected, floral dimensions were strongly positively correlated in both generations (**Supplementary Table 2**). For this reason, we applied principal component analysis to the four flower size measurements (corolla width and length, anther and pistil lengths) and found that PC1, which accounts for 77% of the variation, is a simple average of floral dimensions (loadings reported in **Supplementary Table 3**). PC2 is determined (mainly) by the exertion of both the stigma and anther from face of the flower, while PC3 measures stigma-anther separation. For subsequent tests of trait divergence, we analyzed PC1 as “flower size” and PC2 as “stigma-anther exertion.” For simplicity, we retain the difference (pistil length – anther length) as the trait “stigma-anther separation”. The stigma-anther separation value which had been calculated prior to the PCA exhibits a 0.96 correlation with PC3.

### Divergence among experimental and ancestral populations

All populations diverged from the Ancestor in one or more traits, and for some traits (e.g., Leaf width), almost all populations differed from the Ancestor (**Supplementary Table 1**). More importantly, there was a clear effect of the mating system treatment on the pattern of divergence (**Supplementary Table 4**). The nature of this response differed among traits, with four qualitatively distinct outcomes. The first kind of response, illustrated by days to flower (**Figure 2**, left) and stigma-anther separation (**Figure 2**, right), is where mating systems show consistent differences in trait means that were maintained across both generations 10 and 11. Mean days to flower evolved to the lowest values in the outcrossing populations followed by the mixed mating and selfing populations. Importantly, creating outbred plants in generation 11 within the mixed mating and selfing populations did not alter the relative means of each treatment. This indicates a response driven by allele frequency change (adaptation) and not simply increased homozygosity in the inbreeding populations. Stigma-anther separation showed the opposite pattern to days to flower (the greatest change was in the selfing populations, the least in the outcrossing populations), but the pattern of differences was similar between generations 10 and 11. A previous study showed that both pistil length and anther length exhibit inbreeding depression in this population (Arathi and Kelly, 2003). However, each trait is reduced to a similar extent and so stigma-anther separation, which is the difference (pistil minus anther), has minimal inbreeding depression. In this situation, we predict minimal change from generations 10 to 11, as is observed in **Figure 2**, right.

The second type of response is where differences among treatments evident in generation 10 evaporate in generation 11. This was observed for flower size (**Figure 2**, middle) and reproductive capacity (**Figure 3**, middle). In generation 10, flower size and reproductive capacity were significantly reduced in the inbreeding populations relative to the outbred, but the absence of differences in generation 11 implicates homozygosity and not evolution as the cause.

The third sort of response is non-significance in generation 10 but significance in generation 11. Both measures of autogamy (whether a plant sets any self-seed at all and the number of seed if it succeeds in selfing; **Figure 3**) exhibited this pattern, as did leaf width (**Supplementary Table 4**). For these traits, the differences among mating systems only becomes clear when comparing outbred plants (generation 11) which implies that adaptation and inbreeding depression are having conflicting effects on trait means in generation 10. Plants in the mixed mating and selfing treatments did adapt to become more efficient at selfing, but this improvement is obscured by the general reduction in vigor (and thus in seed production) caused by higher homozygosity. The last category – no difference among mating systems in either generation – was observed only for stigma-anther exertion (**Supplementary Table 4**). Finally, the variance among populations within treatments was always highly significant, usually greatest in Selfing treatment (**Figures 2** and **3**, **Supplementary Table 5**).

The treatment effects evident in **Figures 2** and **3** are based on phenotypic measurements from plants grown simultaneously. We can analyze generations 10 and 11 simultaneously if genetic effects can be distinguished from seasonal differences between generations 10-11. To enable this discrimination of effects, we grew a collection of Generation 10 plants alongside the outbred plants in Generation 11. As a consequence, many genotypes, including the ancestral population, were measured in all four grow-ups (two cohorts within each generation). We use a regression model to distinguish inbreeding effects (present in the mixed mating and selfing populations, but not in the outcrossing or ancestral populations) from cohort-specific environmental effects. The full set of regression coefficients is reported as **Supplementary Table 6**. **Figure 4** illustrates this model fit for a collection of trait pairs. The change from ancestor (purple symbol in **Figure 4**) to the Generation 10 genotypic mean (red, blue, green circles in **Figure 4**) reflects the combined effect of adaptation and inbreeding depression. The phenotypic “release” from inbreeding depression is the transition from Generation 10 to Generation 11 (arrows to x’s in **Figure 4**). There is no release for the outbred populations because the inbreeding level does not change from generation 10 to 11 (circles and x’s are coincident for these populations).

For most traits, there was for a reduction in trait means from ancestral to Generation 10 populations. For example, both leaf width and reproductive capacity declined but to varying extents with mating system. Leaf width declined most severely in the outbred populations, reproductive capacity in the selfing treatment (**Figure 4A**). The transition to outbred plants largely reverses change in reproductive capacity (significant in Generation 10 but not 11) but exaggerates mating system differences in Leaf width (non-significant in Generation 10 but significant in 11). Flower size exhibits a trajectory similar to leaf width (**Figures 4B, D**). Days to flower (**Figure 4C**) and stigma-anther separation (**Figure 4B**) yield more complicated patterns with the inbreeding release apparently acting in different directions in the selfing and mixed mating treatments. However, inbreeding effects were small for both traits. The major exception to trait declines was autogamy (**Figure 4D**). Inbreeding depression causes declines of means in the treatments where selfing is most important to reproductive success. Adaptation is only evident after comparing outbred plants across treatments.

## Discussion

Evolution can occur rapidly when plant populations experience a disruption of pollinator services (Acoca-Pidolle et al., 2023; Bishop et al., 2023) and here we used experimental evolution to explore this process. Mating system varies among *M. guttatus* populations, but the ancestral population for our study (Iron Mountain) is predominantly outcrossing. The fraction of seeds produced by outcrossing (t) has been estimated in each of four distinct field generations at Iron Mountain: t = 0.91 in 1989, t = 0.76 in 1990, t = 0.90 in 2013, and t = 0.91 in 2014 (Willis, 1993; Monnahan et al., 2021). Based on these estimates, we predicted that the mixed and selfing populations of this experiment would show the most substantial trait changes (as they did) because a reduction in t down to 0.1 (our mixed mating treatment) or 0.0 (our selfing treatment) is a major alteration relative to the ancestral environment. Importantly however, the genetic basis of these changes, whether due to adaptation or increased homozygosity, differed among traits.

The magnitude of trait changes over only ten generations is surprisingly large. Stigma-anther separation was reduced by an average of 20% in the mixed mating and 26% in the selfing treatments (**Figure 3**). Autogamy, the number of seeds produced by the 4^th^ flower when left alone, changed markedly in all three mating system treatments, although in different directions. Ancestral plants produced an average of 20.3 seeds by autogamy. In the Mixed mating and Selfing populations, the means increased to 32.3 and 37.9, respectively (an inflation of 59-86%), perhaps reflecting increased allocation to female function (Harder and Barrett, 2006). In contrast, the outcrossing mean (10.3) was half that of Ancestral plants. This suggests rapid evolutionary atrophy of the ability to self when reproduction occurs exclusively by outcrossing. Given rapid atrophy when there is no selfing, the observed ≈10% selfing rate in nature is indicated as an important selection pressure maintaining the population as mixed mating.

The immediate evolutionary response to an environmental change depends on the recruitment of standing genetic variation within a species (Barrett and Schluter, 2008; Bitter et al., 2019). The amount of change in trait means routinely documented by experimental evolution illustrates the evolutionary potential of standing variation. Outside the laboratory setting, resurrection experiments applied to natural plant populations have demonstrated the effective recruitment of standing variation in response to pollinator disruptions (Acoca-Pidolle et al., 2023; Bishop et al., 2023). The magnitudes of changes documented by both field and laboratory experiments are smaller than those that distinguish outcrossing and selfing sister species, the so-called selfing syndrome (Ornduff, 1969; Sicard and Lenhard, 2011). However, this difference may reflect limits on time (the duration of experiments) more than shortages of standing variation.

We included the mixed treatment in this experiment because many natural populations are likely to experience a partial, but not complete, loss of pollinators (Potts et al., 2010; Ramos-Jiliberto et al., 2020; Smith et al., 2022). Mixed mating is a very common reproductive strategy across flowering plants (Johnston et al., 2009). Populations in our mixed mating treatment usually evolved trait values intermediate to the outcrossing and selfing populations (**Figures 2** and **3**). This is not surprising. Predicting response to selection from genetic variance components is quite complicated with inbreeding (Cockerham and Weir, 1984; Kelly, 1999c), but for most genetic models, mixed mating is likely to produce an intermediate response in the short term. However, this may change over slightly longer time scales as genetic variances change owing the combined effects of selection and inbreeding.

A serious limitation for purely selfing populations is that every individual is a distinct lineage, genetically isolated from all other lineages. Even a low level of outcrossing allows alleles to be shared between lineages, which allows beneficial alleles to be concentrated into the same genetic background (Cooper, 2007). For this reason, a low level of outcrossing could accelerate adaptation to efficient self-fertilization relative to a population compelled to complete selfing. After 10 generations of selection, our selfing populations were slightly advanced of our mixed mating populations *on average* in terms of autogamy (**Figure 3**). However, some of the mixed populations clearly exceed some of the selfing populations, particularly in terms of the seed number component. Response to selection in the pure selfing populations will plateau as a single lineage (or a small number of fitness-equivalent lineages) become predominate. The “allele sharing” enabled by mixed mating could allow a more sustained response over longer time scales.

### Inbreeding depression versus adaptation

To understand the results, it was essential to experimentally disentangle adaptation from inbreeding depression (**Figure 4**). Inbreeding depression is usually considered in relation to fitness and attributed to either deleterious recessive or partially recessive alleles or to overdominance (Charlesworth and Charlesworth, 1987; Dudash and Carr, 1998). Variation in quantitative traits like flower size or phenology may not be caused by rare partially recessive alleles or by overdominance, but that does not imply that they are unaffected by inbreeding. Any quantitative trait locus with dominance (partial or complete) will cause the mean of a trait to change with inbreeding (Lynch and Walsh, 1998). In fact, all else equal, intermediate frequency alleles generate much more inbreeding depression than rare alleles (Kelly, 1999a). In quantitative genetics, this effect is usually termed ‘directional dominance’ instead of inbreeding depression because trait changes are not always negative. Regardless, the trajectories of **Figure 4** illustrate how adaptation and inbreeding can drive phenotypic changes in interestingly different ways. In some cases, such as the probability of setting seed by selfing, they are strongly antagonistic. Beyond the present experiment, the conflation of inbreeding depression with adaptation is a concern for any study comparing trait differences among populations that differ in outcrossing rate, either in space or through time.

The trait differences in generation 11 are easier to interpret genetically, they are not more biologically important than the differences evident in generation 10. From a demographic perspective, it is clearly important that pollinator loss increases homozygosity as well as spurring adaptation. Inbreeding depression can greatly reduce absolute individual fitness and thus population viability. Regarding evolution, while the crosses of generation 11 disentangled inbreeding from adaptation, these processes certainly interacted to determine trait changes over the course of the experiment. Selection acts on genetic variation in a different way in outcrossing populations, where allelic effects are an average across heterozygous and homozygous states, than in selfing populations where homozygous effects dominate. Inbreeding also accelerates the stochastic loss of genetic variation due to genetic drift and linked selection (Caballero and Hill, 1992; Caballero and Santiago, 1995; Pekkala et al., 2014; Busch et al., 2022). These processes can cause deleterious alleles, initially at low frequency, to reach fixation within a population. This “fixed load” (Willi et al., 2013) is a consequence of allele frequency change and is not revealed by crosses within a population (as in our contrast of plants from Generations 10 and 11). However, this process accelerates differentiation among replicate populations.

### Multivariate evolution in response to pollinator loss

Given that autogamy was a key fitness component in this experiment, rapid evolution was expected. We had also anticipated that stigma-anther separation would decline in selfing and mixed mating treatments – a shorter distance between the anthers and stigmatic surface can increase the probability of pollen transfer (Carr et al., 1997; Carvallo and Medel, 2010; Opedal, 2018). It may also increase “delayed self-pollination” (Goodwillie and Weber, 2018), where self-pollination is deferred to the end of a flower’s lifetime. This is relevant given that Iron Mountain *M. guttatus* exhibits delayed selfing in a quantitative fashion (Arathi and Kelly, 2004). Across the genus, selfing species routinely have reduced stigma-anther separation relative to outcrossing sister species, e.g. *M. nasutus* (Fishman et al., 2002), *M. micranthus* (Carr and Fenster, 1994), *M. platycalyx* (Dole, 1992), and *M. cupriphilus* (Macnair, 1989).

The selection responses for other floral traits to pollinator loss/reduction were harder to anticipate. Flower size is typically reduced in selfng species. This may be driven by energetics: Why invest in expensive flowers when attracting pollinators is not required (Cruden and Lyon, 1985)? Alternatively, changes in corolla morphology may be essential to facilitate self-pollination. We observed reductions in flower size in the treatments with selfing, but differences in the means of each treatment group were mostly due to inbreeding depression (**Figure 2**). The genetic correlation between flower size and days to flower was likely a key factor here. Genotypes that rapidly progress to flower tend to have smaller flowers than genotypes that develop more slowly (Kelly, 2008). Our experimental design (hand-pollination done only on Day 25 in the Outcrossing treatment) clearly imposed selection for rapid progression to flowering and there was a strong response (**Figure 2**), which produced a correlated response to selection towards smaller flowers in the outcrossing populations. This correlated selection should have been weaker in the Mixed mating and mostly absent in the Selfing treatment populations. Interestingly, all experimental populations evolved smaller flowers relative to the Ancestors (dotted line in **Figure 2**), although perhaps under different pressures.

Studies of contemporary evolution, in the field or greenhouse, can address questions about the ‘order of events’ in the evolutionary transition from outcrossing to selfing. The selfing syndrome consists of traits that range from the direct act of reproduction (e.g. the number of seeds per flower when there is no visitation) to traits affecting that act (e.g. herkogamy and dichogamy) to traits affecting the likelihood of outcrossing as opposed to self-fertilization (e.g. attraction or reward of pollinators) to traits that affect the overall efficiency of reproduction under different mating systems (e.g. pollen/ovule ratios, flowering time). Should these various traits evolve simultaneously or sequentially? It is natural to think that the traits most closely linked to reproduction would evolve first followed by changes in ‘secondary’ characteristics that facilitate attraction of pollinators. Contrary to this view, flower size and floral rewards have shown immediate changes in field populations (Acoca-Pidolle et al., 2023; Bishop et al., 2023), simultaneous with changes in the selfing rate. In this study, we see substantial change not only in the primary traits (autogamy and herkogamy), but still also in the secondary traits like flower size and days to flower (**Figures 2** and **3**). Current evidence thus suggests that pollinator loss will result in a rapid and multivariate response to selection.

## Data Availability

The original contributions presented in the study are included in the article/**Supplementary Material**. Further inquiries can be directed to the corresponding author.
