# Supplementary material for: Experimental evolution suggests rapid assembly of the ‘selfing syndrome’ from standing variation in Mimulus guttatus
Source: Front Plant Sci. 2024 Aug 27;15:1378568. doi: 10.3389/fpls.2024.1378568 (PMC11388319; doi:10.3389/fpls.2024.1378568)
Supplement: Supplementary file 7 [file DataSheet1.pdf]

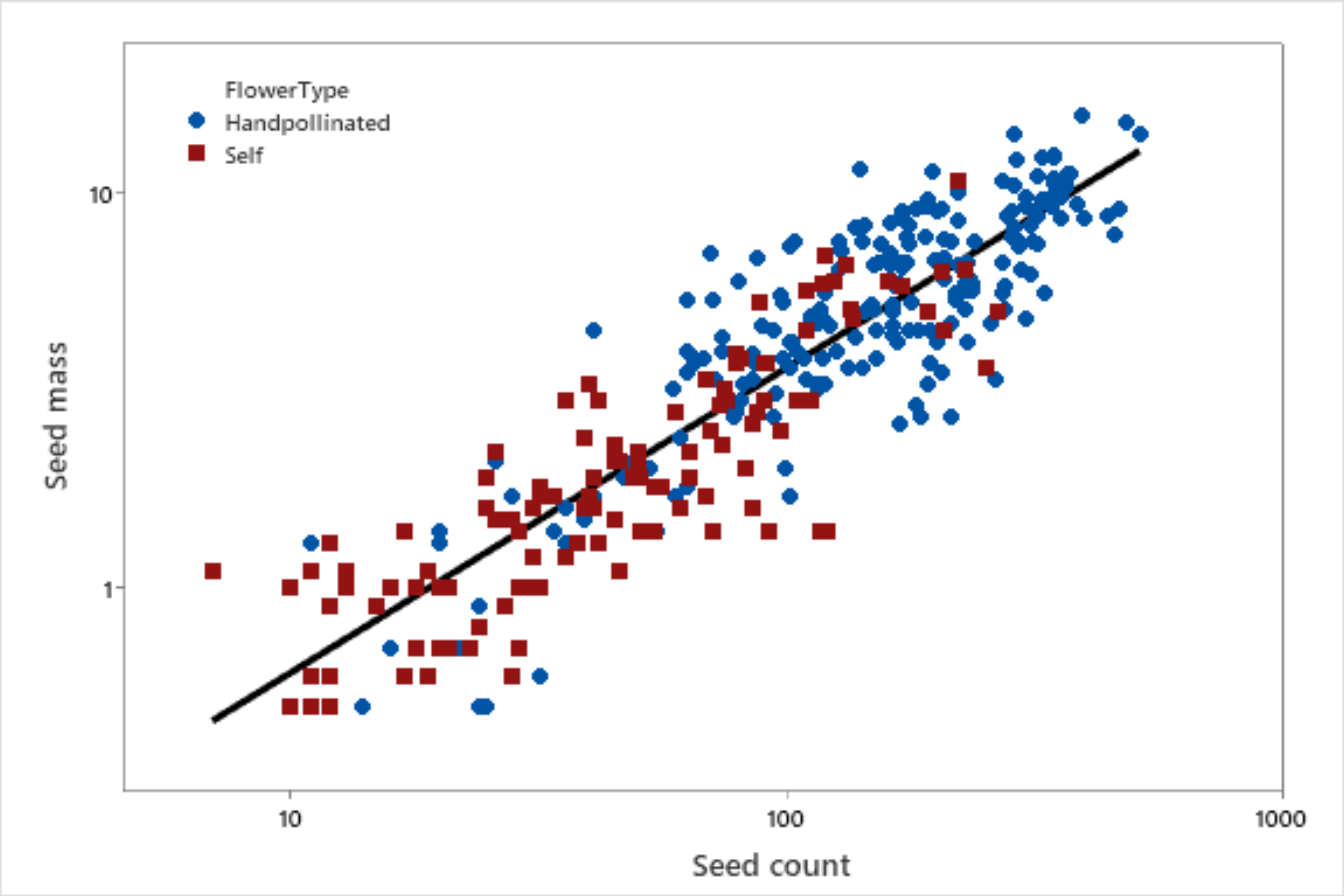

Supplemental Figure A. The allometric relationship between seed mass (in mg) and seed count. $Log_{10}(mass) = -0.9915 + 0.7749 Log_{10}(count)$. Since the slope < 1, individual seed mass declines as seed number per flower increases. n = 324 (both types of flowers combined).
